# Overview. Preterm labour: mechanisms and management

**DOI:** 10.1186/1471-2393-7-S1-S2

**Published:** 2007-06-01

**Authors:** Andrés López Bernal

**Affiliations:** 1 University of Bristol, Clinical Science at South Bristol (Obstetrics and Gynaecology), St Michael's Hospital and Dorothy Hodgkin Building, Whitson Street, Bristol, BS1 3NY, UK

## Abstract

Preterm birth remains a major cause of perinatal mortality and long term handicap in surviving infants. This is one of the most important clinical problems in Europe and across the world. While some preterm births are iatrogenic, associated with severe complications of pregnancy (e.g. hypertensive disorders, antepartum haemorrhage, infection), or the result of multiple pregnancies following assisted reproduction, a high proportion of preterm births occur following spontaneous preterm labour of unknown cause. Early intervention in this group of women would have a significant impact on neonatal mortality and morbidity figures. However, the endocrine changes preceding parturition in women remain elusive and this makes it difficult to predict spontaneous labour at term, let alone preterm labour. Moreover our understanding of myometrial physiology remains rudimentary, limiting our options to devise improved pharmacological strategies to control uterine contractility when this is indicated. There is a need for concerted European and international research efforts to improve our knowledge of the mechanism of labour in women, to identify diagnostic markers to predict preterm labour and to develop uterine selective drugs to inhibit uterine contractions in a safe and efficient manner. This aim will be achieved by multidisciplinary research efforts from academics and industry, using traditional laboratory and clinical research methods, as well as novel technologies.

## The problem of preterm labour

The factor(s) controlling the spontaneous onset of labour are not known. This is frustrating from a physiological point of view, and it is also a major clinical problem. Spontaneous preterm deliveries (before 37 completed weeks of gestation) account for 10% of all births and yet they account for 75% of neonatal deaths. Thus alterations in the timing of the onset of labour *per se *(excluding congenital malformations and elective preterm deliveries for severe complications of pregnancy) are a major contributor to perinatal mortality [1,2]. The last trimester of pregnancy is necessary for the maturation of the fetal lungs and other organs in preparation for extrauterine life. If this process is interrupted by an early delivery the chances of survival of the newborn are severely decreased. The mortality rate is higher at lower gestational ages. For example it increases from 2 ‰ (two per thousand deliveries) at 37–40 weeks, to 18 ‰ at 32–36 weeks and 216 ‰ at 24–31 weeks [3]. Despite considerable improvements in special care baby units the perinatal mortality rates in the UK remain steady, and there is a wide range of both short-term and long-term morbidity and handicap in the surviving infants [4,5]. The challenges of extreme prematurity often make international headlines in the Western media, but prematurity affects both rich and developing countries.

The uterus is a myogenic organ and it contracts spontaneously following waves of electrical activity that result in membrane depolarization, a rise in intracellular calcium and the generation of force. While uterine activity can occur in the absence of hormonal or neural stimulation, the activation of a number of G protein coupled receptors (GPCRs) present on myometrial cells has profound stimulatory or inhibitory effects on contractions. For example, receptors coupled to Gα_q _e.g. oxytocin receptors (OXTR), endothelin-receptors (EDNRA), some prostanoids receptors (PTGER1, PTGFR, TBXA1R), stimulate contractility by activating the phospholipase C/Ca^2+ ^pathway; receptors coupled to Gα_s _e.g. β_2_-adrenoceptors (ADRB2), prostanoid PTGDR, PTGER2 and PTGIR relax the uterus by stimulating adenylyl cyclase (ADCY) and increasing myometrial cyclic AMP levels; and receptors coupled to Gα_i _e.g. α_2_-adrenoceptors (ADRA2), muscarinic receptors (CHRM), potentiate contractility, probably by inhibiting cyclic AMP production [6]. The uterus responds to many agonists, hence changes in the level of receptor expression and coupling to intracellular signalling pathways are likely to be involved in the regulation of uterine contractility. Uterine quiescence during pregnancy and the increased activity associated with the spontaneous onset of labour are likely to be reflected by changes in myometrial receptor function [6,7].

## Endocrinology of parturition

For many years the overwhelming candidate responsible for pregnancy maintenance has been progesterone. This is based on the fact that in mammals the onset of labour is associated with mechanisms that result in maternal progesterone withdrawal. In the sheep parturition is initiated by activation of the fetal pituitary-adrenal axis [8], with increased fetal cortisol secretion [9], followed by the activation of placental cytochrome P450 (CYP17A family) enzymes with 17α hydroxylase and 17–20 lyase activities [10]. As a consequence of this glucocorticoids-dependent enzyme activation there is increased conversion of C19- to C18-steroids, so that maternal progesterone levels fall and oestradiol levels rise [8]. These steroid changes promote increased intrauterine production of prostaglandins, cervical softening and uterine contractions. Moreover, fetal adrenal cortisol induces prostaglandin synthase type 2 in placental trophoblast with an increase in prostaglandin E2 production which reinforces the activation of the P450 cascade [11]. In corpus luteum-dependent species (goats, rabbits, rats, mice), the onset of labour is triggered by the release of prostaglandin F2α from the endometrium leading to the demise of the corpus luteum. Luteolysis is mediated by activation of prostaglandin F receptors (PTGFR) [12] and provokes a fall in maternal progesterone levels, which is rapidly followed by the onset of labour.

The factors responsible for parturition in women remain unknown and the endocrine paradigms described above do not fit primates. Progesterone production from the corpus luteum is essential in early human pregnancy, but the organ is not required for the last two thirds of gestation when progesterone production is taken over by the placenta [13]. Moreover, there is no induction of placental CYP17A enzymes by cortisol around the time of parturition and the levels of progesterone and oestrogens remain stable. Progesterone is metabolised by 20α-hydroxysteroid dehydrogenase in uterine tissues [14] but there is no conclusive evidence that the activity of this enzyme alters the local oestrogen/progesterone ratio before parturition.

The fetal pituitary-adrenal axis, which is very active in late gestation, appears to have only a supportive role in human parturition. Observations in anencephaly pregnancies with no residual pituitary/adrenal function showed that the mean length of gestation was similar to that in women carrying normal singleton fetuses, but with a much wider scatter around the mean [15]. This suggests that normal fetal pituitary adrenal function is required for the fine-tuning of the timing of parturition but that spontaneous labour can still occur in anencephaly. In primates, the fetal adrenal produces large amounts of androgen precursors, notably dehydroepiandrosterone sulphates, which are converted to oestrogens in the placenta by sulphatase and aromatase activities. Remarkably, normal pregnancy and parturition have been reported in patients with placental sulphatase or aromatase deficiencies with very low levels of oestrogens [16]. Thus, in women changes in circulating steroid levels are not a pre-requisite for the onset of labour.

Nevertheless our understanding of steroid action in the pregnant uterus is incomplete and further research in this area will be beneficial. It has been proposed that parturition in women results from coordinated functional progesterone withdrawal and oestrogen activation in myometrial tissue [17,18]. The progesterone receptor (PGR) is a ligand-activated transcription factor of the steroid receptor superfamily. Human progesterone receptors exist in three isoforms originating from the same gene, a full length 116 kDa PGR-B and two smaller N-terminally truncated forms, a 94 kDa PGR-A and a 60 kDa PGR-C [19,20]. Binding of progesterone to PGR-B results in activation of progesterone-responsive genes, however in myometrial cells the action of PGR-B is antagonised by PGR-A which acts as a dominant repressor of transcription [21]. Some studies suggest that the relative abundance of PGR-A over PGR-B in human myometrium increases in labour, leading to functional progesterone withdrawal [21,22]. This switch is accompanied by an increase in oestrogen receptor transcripts [22] which potentially could result in increased oestrogen responsiveness. It is not known what brings about the changes in PGR expression in the uterus at the time of parturition; however it is possible that these are mediated by changes in histone acetylase activity which is required for PGR transcription. In mice the use of a histone deacetylase inhibitor delayed significantly the onset of labour [23]. Recent data suggest that labour is associated with an increase in PGR-C isoforms, probably mediated through the NFKB pathway [24]. PGR-C reside mainly in the cytosol of myometrial cells and it is thought that the increase in PGR-C/PGR-B ratio takes progesterone away from PGR-B leading to low PGR binding to nuclear chromatin in myometrium in labour [24,25]. The concept that PGR-B, as opposed to PGR-A or PGR-C, is essential to progesterone action in myometrium appears to be challenged by experiments in transgenic mice that show that PGR-A and PGR-B have overlapping, rather than antagonistic actions in fertility and uterine function [26]. Moreover the functional role of PGR isoforms may be dependent on the availability of specific co-regulators such as the cAMP-response element-binding protein (CREB)-binding protein [23] the steroid receptor coactivators 2 and 3 and [23,27] and the protein-associated splicing factor SFPQ [28]. Experiments in pregnant rats have shown that SFPQ inhibits the transcriptional activity of PGR by a mechanism that involves blocking nuclear progesterone response elements and by promoting increased catabolism of PGR [28]. It will be of interest to study these mechanisms in human pregnant myometrium near term.

A new approach to our understanding of progesterone function has followed the description of membrane-bound isoforms of the PGR (mPGR) resembling GPCRs in human myometrium [29,30]. The signalling pathway for mPGR has been investigated in human myometrial cells and the evidence suggests that the receptor stimulates mitogen-activated protein kinases (MAPK) through a Gi dependent mechanism. Moreover activation of mPGRs leads to inhibition of cAMP synthesis and phosphorylation of MYL by MAPK [29]. This poses the challenging hypothesis that progesterone may participate both in pregnancy maintenance and in the stimulation of myometrial contractility at the onset of labour through the interplay of intracellular PGR receptors and Gi-coupled mPGR proteins. Further research into the functional role of the different PGR proteins in myometrium will be of great interest.

## Gene regulation

There is very little information on the regulation of genes that promote uterine relaxation in pregnancy. An important concept is that uterine relaxation in pregnancy may be due to lack of electrical and metabolic co-ordination between myometrial smooth-muscle cells. Gap junctions are specialized protein channels that facilitate the propagation of electrical activity and the exchange of small molecules between cells. Thus, the appearance of gap junctions in myometrium is thought to herald the onset of labour in animals and humans [31-33]. GJA1 (connexin 43) is one of the main structural proteins in myometrial gap junctions; its expression is stimulated by oestradiol and inhibited by progesterone [34]. Detailed studies of *GJA1 *gene regulation indicate that the family of activator protein-1 transcription factors is critical to determine the level of GJA1 expression in myometrial cells [35,36]. Retinoic acid upregulates GJA1 expression in human endometrial and myometrial cells probably through nuclear receptors [37,38]. A number of protein kinases are involved in the functional regulation of GJA1, including PRKA, PRKC and mitogen-activated protein kinase 1 (MAPK1). Phosphorylation at Ser-368 seems important for translocation of GJA1 from the cytosol and its assembly on the plasma membrane [39]. On the other hand phosphorylation of GJA1 and other connexins may also trigger internalization and degradation [40]. In this regard it is interesting to note that activation of MAPK1 in pregnant rat myometrium leads to phosphorylation of GJA1 at Ser-255 and this causes a loss of amplitude and synchronization of uterine contractions [41]. In mice, conditional deletion of the GJA1 gene causes a dramatic delay in parturition [42]. A better understanding of the pathways that regulate GJA1 expression and phosphorylation in human myometrium should clarify the role of these proteins during pregnancy and labour.

Human chorionic gonadotrophin (hCG) has an essential role in prolonging the life of the corpus luteum to allow the establishment of pregnancy and its continuation through the first trimester. There is some evidence that in addition to its effect on the ovary hCG has a direct effect on myometrial smooth muscle to promote uterine quiescence. Receptors for hCG (LHCGR) have been described in human pregnant and non-pregnant myometrium; interestingly the binding of hCG to myometrial tissue decreases during term and preterm labour [43]. The application of hCG to human myometrial strips obtained from late pregnant women inhibits contractility [44] and this raises the intriguing possibility that placental gonadotrophin is an endogenous tocolytic agent. The action of hCG involves positive coupling to ADCY through LHCGR-Gαs and inhibition of intracellular Ca^2+ ^fluxes through a cAMP/PRKA mechanism [45]. It is no clear what targets are phosphorylated by PRKA upon stimulation of the LHCGR but the available evidence suggest that hCG antagonises proteins involved in oxytocin signalling [45]. Moreover, activation of LHCGR in human myometrial cells leads to loss of gap junction formation and downregulation of GJA1 protein by a PRKA mediated effect [46]. Another mechanism by which the LHCGR can promote uterine quiescence is through inhibition of the phosphodiesterase PDE5 gene in human myometrial cells, thus potentiating the effect of cyclic nucleotides [47].

Recent data suggest that the regulation of the LHCGR gene in human myometrium involves the binding of specificity protein (Sp)-like transcription factors to the proximal promoter region of the gene. SP1, SP3 and SP4 proteins are involved in recruiting histone deacetylases to the promoter, blocking transcriptional activation by preventing chromatin remodelling [48]. A previous report had suggested that parturition in women and mice is associated with a loss of co-activators containing histone deacetylase activity in the uterus [23]. SP proteins are activated by PRKA [49] providing a feedback mechanism by which cAMP can regulate the level of LHCGR expression. Moreover there is some evidence that hCG may be involved in the upregulation of Gαs protein observed in human myometrium in pregnancy [50]; the transcriptional activity of the Gαs promoter in human myometrial cells is modulated by hCG in a biphasic manner by a mechanism that appears to involve PRKA activation of SP1 [51].

## The management of preterm labour

The most commonly used drugs for the treatment of threatened preterm labour have been the betamimetics. Ritodrine and terbutaline were introduced as selective β_2_-adrenoceptor (ADRB2) agonists and in 1980 the FDA approved ritodrine hydrochloride for use in preterm labour. ADRB2 receptors are positively coupled to Gαs/ADCY and their effects are mediated by the cAMP pathway; although the exact mechanism of action is not known it is likely to involve activation of PRKA and phosphorylation of proteins involved in smooth muscle relaxation. Betamimetics are effective at delaying delivery in women in preterm labour for 48 hours, but there is no evidence that this relatively short delay provides any benefit in terms of perinatal mortality or morbidity [52]. Stimulation of ADRB2 receptors results in desensitization and downregulation. Short term tocolysis with intravenous ritodrine may be helpful to transfer women in preterm labour to a hospital with better neonatal facilities or to allow time to complete a course of antenatal glucocorticoids, but even these advantages are controversial. Long term tocolysis with oral terbutaline for maintenance therapy after threatened preterm labour shows no benefit compared to placebo [53]. The use of ADRB2 agonists is associated with unpleasant and potentially serious cardiovascular, metabolic and neuromuscular side effects and this represents a major drawback to their clinical use. As reported in this issue, new data show that the predominant β-adrenoceptor in late pregnant human myometrium is the β_3_-adrenoceptor (ADRB3) rather than the ADRB2 [54]. Stimulation of ADRB3 relaxes pregnant myometrial strips probably by opening potassium channels [55]. It will be of interest to explore the development of specific ADRB3 agonists as potential tocolytic agents.

Oxytocin has a prominent role in promoting strong uterine contractions in the later stages of labour and in the immediate puerperium, and in facilitating milk ejection during lactation. The concentration of myometrial OXTR is very high in late pregnancy, making the uterus very sensitive to oxytocin [56-58] and providing a relatively selective pharmacological target [59]. In 2000 atosiban became the first oxytocin antagonist specifically approved for the management of preterm labour. Atosiban is a competitive inhibitor of the OXTR and blocks OXT-induced Ca^2+ ^increase in myometrial cells in a reversible manner [60]. However atosiban has a high affinity for the vasopressin AVPR1A receptor and this may result in unwanted side effects. Clinical trials with atosiban are encouraging but inconclusive; most studies have compared its tocolytic efficacy with that of betamimetics and the results show that it is at least as efficient and with a much lower frequency of side effects [61,62]. A new generation of OXT antagonists includes barusiban which is more potent than atosiban and very selective for OXTR versus AVPR1A receptors [63]. Other approaches include the development of non-peptide orally active OXTR antagonists [64,65]. The potential of these drugs for the management of preterm labour should be investigated with appropriate clinical trials.

Progress in the management of preterm labour has been hampered by our lack of understanding of the process of parturition in humans and by the unavailability of drugs capable of inhibiting uterine contractility efficiently, without causing potentially serious side effects for the mother or the baby. Conventional tocolytic agents e.g. β_2_-adrenoceptor (ADRB2) agonists, calcium channel antagonists, prostaglandin synthesis inhibitors), all lack uterine selectivity. The effectiveness of oxytocin antagonists is not proven. New approaches to develop better drugs for the management of preterm labour discussed in this issue include studies on prostaglandin PTGFR receptor antagonists, and phosphodiesterase (PDE4) inhibitors. More research is necessary to identify the physiological trigger for parturition and to understand why the process may be activated prematurely in some pregnancies. It is also necessary to investigate the biochemical mechanisms regulating uterine smooth muscle activity in order to develop more effective and selective therapy to control uterine contractions when this is indicated. Parturition is a complex physiological event involving integrated functional responses in several maternal and fetal organs. It is likely that broad genomic and proteomic approaches will highlight relevant mechanisms, however I believe it is important to focus on improving our understanding of the regulation and signalling pathways of receptors whose influence on uterine contractility is well established.

## The need to promote research into preterm labour

The quality of research into human parturition and preterm labour in Europe is high, attested by the continuing presence of European speakers at the major North American and other international obstetric and perinatal scientific meetings and by the regular appearance of excellent manuscripts from European laboratories in top peer-reviewed journals. However this high quality output reflects rather fragmented research efforts from enthusiastic groups that work in relative isolation. There is a need for concerted research efforts from different European laboratories to make substantial contributions towards solving the problem of preterm labour. This will require traditional physiological and biochemical studies as well as incorporating modern genomic and proteomic approaches and new imaging techniques, electromyography, bioengineering and nanotechnology. Moreover the development of any new diagnostic methods or uterine-selective drugs must be validated with appropriately designed clinical trials. The European Commission continues to promote high quality research to improve the health and wellbeing of European citizens and those in underprivileged countries of the world. A comprehensive FP7 funding programme has just been launched. The meetings in Tarragona, and subsequent meetings in London and Paris have resulted in the creation of a European Preterm Labour Group of investigators to cement collaboration and publicise the need to invest research funds into a better understanding of human parturition and preterm labour, so that most women can benefit from a successful pregnancy and a healthy newborn.

## Competing interests

The author declares that they have no competing interests.
